# Efficacy and Safety of Oral Green Tea Preparations in Skin Ailments: A Systematic Review of Clinical Studies

**DOI:** 10.3390/nu14153149

**Published:** 2022-07-30

**Authors:** Antonella Di Sotto, Marco Gullì, Ester Percaccio, Annabella Vitalone, Gabriela Mazzanti, Silvia Di Giacomo

**Affiliations:** Department of Physiology and Pharmacology “V. Erspamer”, Sapienza University of Rome, P.le Aldo Moro 5, 00185 Rome, Italy; marco.gulli@uniroma1.it (M.G.); ester.percaccio@uniroma1.it (E.P.); annabella.vitalone@uniroma1.it (A.V.); gabriela.mazzanti@uniroma1.it (G.M.); silvia.digiacomo@uniroma1.it (S.D.G.)

**Keywords:** *Camellia sinensis*, polyphenols, catechins, epigallocatechin-3-gallate, skin, acne, clinical trials, PRISMA, photoaging, safety concerns, efficacy

## Abstract

Green-tea-based products and their polyphenols, especially epigallocatechin-3-gallate, have attracted great attention over the years as possible nutraceuticals, due to their promising bioactivities, especially antioxidant and anti-inflammatory, which could be exploited in several diseases, including skin ailments. In this context, the present study aimed at reviewing clinical evidence about the benefits of the oral administration of green tea preparations and its polyphenols to relieve skin disorders, to point out the current knowledge, and to suggest possible novel strategies to effectively exploit the properties of green tea, also managing safety risks. To this end, a systematic review of the existing literature was carried out, using the PRISMA method. Few studies, including five focused on UV-induced erythema and skin alterations, three on photoaging, two on antioxidant skin defenses, and one on acne and genodermatosis, were retrieved. Despite several benefits, clinical evidence only supports the use of oral green tea preparations to protect skin from damage induced by ultraviolet radiation; in other cases, conflicting results and methodological limits of clinical trials do not allow one to clarify their efficacy. Therefore, their application as adjuvant or alternative sunscreen-protective interventions could be encouraged, in compliance with the safety recommendations.

## 1. Introduction

Originating from China, tea has a long history of use that spread across numerous countries, over thousands of years. Nowadays, it represents one of the most popular beverages in the world, being consumed far ahead of other drinks, such as wine, beer, and coffee, and second only to water [1]. It is estimated that an average of about 120 mL of tea is consumed by a person per day, with higher amounts (about 540 mL per day) in Great Britain, which represents one of the largest consumers worldwide [1]. This interest arises from its nutritional and healing properties, which stimulate research to exploit tea for nutraceutical and pharmaceutical purposes [2].

The tea plant (*Camellia sinensis* (L.) O. Kuntze; Fam. Theaceae) originated in Southeast Asia over 4000 years ago and is currently produced in over 35 countries: its leaves are processed by various techniques to obtain five commonly recognized tea types, including white, green, oolong, black, and pu-erh teas. The processing of tea leaves is based on some major steps, including withering, rolling, fermentation, and drying [3]; among them, the degree and nature of fermentation lead to a different chemical composition of processed leaves, which, in turn, can result in specific nutritional and healing properties of derived beverages [4]. Although black tea is the most consumed worldwide, green tea has attracted great attention over the years, not only as a beverage, being especially used in East Asia, but also as extracts for its promising nutraceutical properties, which increased its popularity and market demand [3,4].

**Figure 1:**
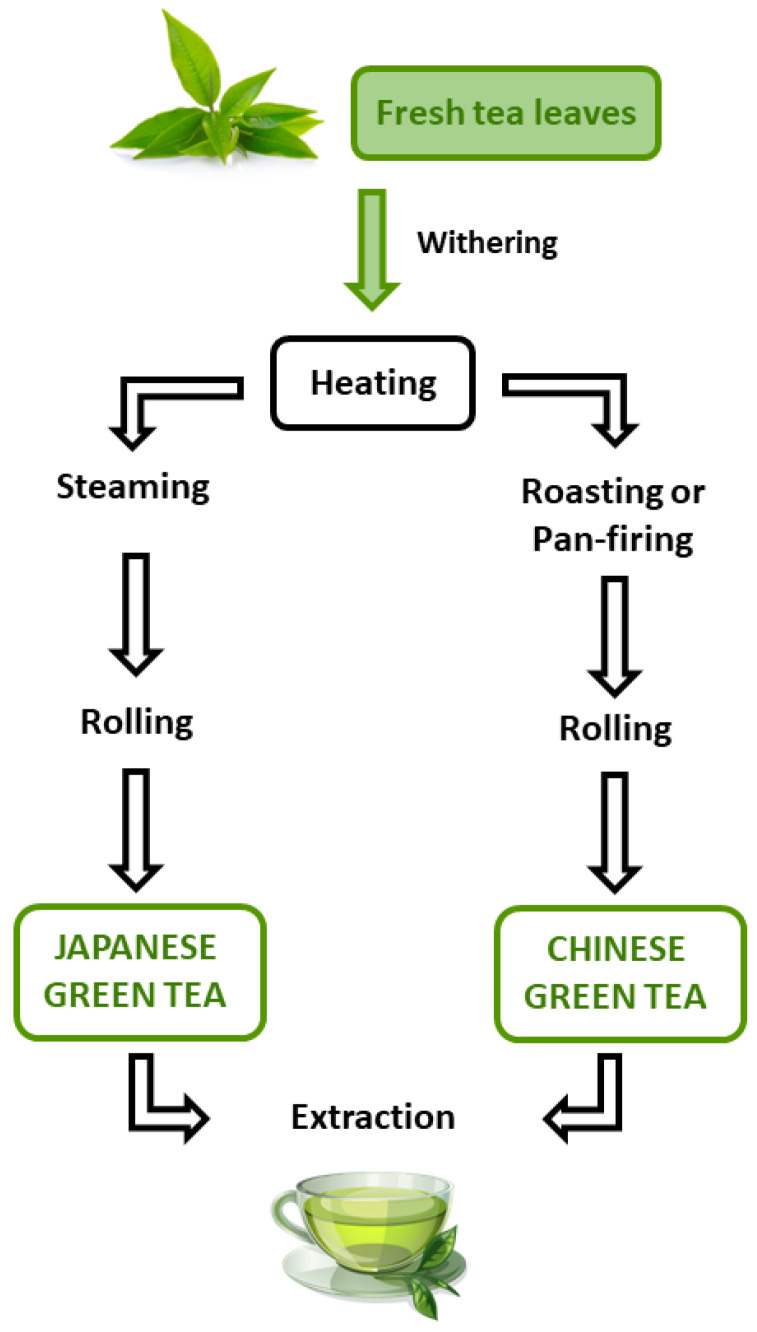
Phases of green tea processing.

Green tea is obtained by heating the fresh leaves of *C. sinensis* in order to inactive oxidative enzymes (i.e., polyphenol oxidase and peroxidase), which are ubiquitously distributed in tea leaves, thus preventing oxidation of polyphenolic components [3]; thereafter, they are subjected to rolling and drying to obtain the final tea product (Figure 1). Different heating methodologies, including steaming (around 100 °C) and roasting or pan-firing (around 300 °C), are applied to obtain Japanese and Chinese green teas, respectively [3]. Lacking fermentation preserves high levels of tea polyphenols, especially catechins (about 60−80% of total amount), and of the amino acid L-theanine, which contribute to the typical astringency, umami, and sweetness aftertaste of green tea [1]. Catechins’ amount can achieve up to 30% of dry weight of green tea, such as fresh tea leaves, but can be decreased due to high-temperature heating or drying steps [3].

**Figure 2:**
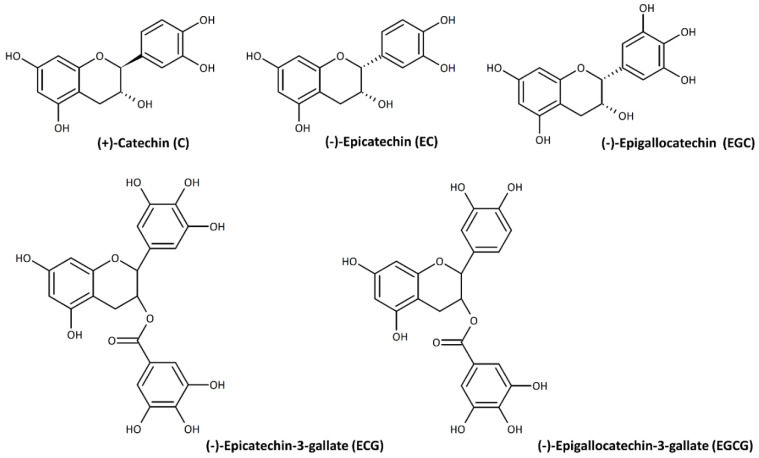
Chemical structures of major tea polyphenols, drawn by ACD/ChemSketch 2018.1.1 software.

The flavanols catechin (C), epicatechin (EC), and epigallocatechin (EGC), and their gallic acid derivatives, i.e., epicatechin-3-gallate (ECG) and epigallocatechin-3-gallate (EGCG), are the major representative green tea polyphenols (GTP) (Figure 2) [1]. The presence of several hydroxyl groups in their chemical structure, which could interact with reactive oxidizing species, is a key feature of their antioxidant activity [5]. Along with catechins, green tea contains a range of other bioactive compounds, including methylxanthines, especially caffeine, polysaccharides, amino acids (i.e., GABA and L-theanine), pigments, and saponins [4].

Green tea consumption has been associated with a reduced risk of cardiovascular diseases in population studies: these benefits seem to arise from the ability of GTP to reduce blood lipids, oxidative stress and inflammation, and to enhance cardiomyocyte and endothelial function [6]. Moreover, green tea supplementation has been reported to improve glycemic control in diabetic patients and healthy subjects and to prevent lipid accumulation, leading to an increased glycolipid metabolism [7]. A reduction in oxidative stress and inflammation seem to mediate the hepatoprotective and neuroprotective effects of green tea and to attenuate symptoms of chronic gut diseases, such as inflammatory bowel disease [7].

Polyphenols are usually recognized to be responsible for the remarkable antioxidant properties of green tea [8], which led to an interest in its health benefits as a nutraceutical and preventive strategy for cardiovascular, metabolic, inflammatory, and degenerative diseases [4,9,10]. Owing to the presence of multiple hydroxylic groups in their chemical structure, these compounds can reduce and quench reactive oxygen species (ROS) and chelate metal ions. GTP can also stimulate endogenous antioxidant defenses, through the activation of the nuclear factor erythroid 2-related factor (Nrf2) pathway and regulate pro-inflammatory and anti-inflammatory signaling and factors, such as NF-κB, TNF-α, Toll-like receptors, and COX-2 [7,11,12,13,14]. Immunomodulatory, genoprotective, and chemopreventive effects, mainly ascribed to the pro-oxidant and anti-proliferative activities of GTP, have been reported too [7,12].

Among GTP, EGCG is the most extensively studied for several possible pharmacological applications, due to its abundance (about 65% of total catechins) and outstanding antioxidant power [11]. EGCG has been reported as able to rescue cognitive deficits in animal models of Alzheimer’s disease, through anti-inflammatory and antioxidant mechanisms, and to prevent the formation of Aβ1-42 oligomers [15]. Accordingly, it exhibited a multitargeted chemopreventive activity in different cancer models, preventing both the development and recurrence of cancer; the effect was found mediated by pleiotropic mechanisms, including antioxidant, anti-inflammatory and detoxifying effects, cell cycle and epigenetic modulation, stimulation of apoptosis, and inhibition of angiogenesis [9]. Furthermore, EGCG induced chemosensitization in all stages of breast and prostate cancer, by receptor-mediated mechanisms; indeed, it antagonized androgen action and decreased androgen receptor (AR) expression in hormone-responsive prostate cancer cells [16]. Meanwhile, it sensitized hormone-responsive breast cancer cells to drugs targeting steroid receptors [17].

**Figure 3:**
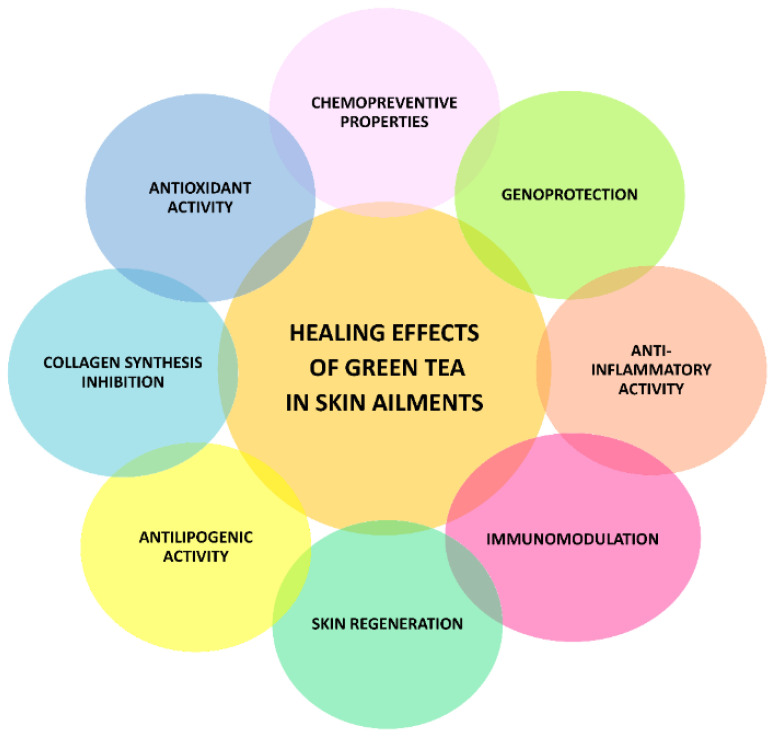
Mechanisms accounting for the healing properties of green tea extracts and polyphenols in skin ailments.

In recent years, many studies have suggested beneficial properties of both topical and systemic application of green tea to ameliorate skin illness (Figure 3). Preclinical studies highlighted the ability of green tea polyphenols to protect skin towards UV-induced damage and immunosuppression [2]. Moreover, GTPs have been shown to possess antimelanogenic properties, mediated by tyrosinase inhibition, and antiphotoaging and antiwrinkle ones [18], likely ascribed to their antioxidant power and to the ability to stimulate collagen and elastin production, while hindering degrading enzymes [19]. EGCG has also been found endowed with chemopreventive effects against UV-induced skin cancer in animal models [20]; furthermore, it activates skin cells in wound healing, likely due to its angiogenetic and antifibrotic properties, leading to tissue remodeling [21]. Other studies [22] also reported that green tea extracts were able to decrease lipogenesis in sebocytes, to promote sebocytes apoptosis, and to inhibit both growth and inflammatory processes associated with *Propionebacterium acnes* infection, thus suggesting an interest in the treatment of acne. Preliminary evidence suggested a possible interest in GTP as antileishmanial strategies too; however, further studies are needed to confirm its efficacy, clinically and in animal models [23].

Preclinical evidence highlighted that both green tea extracts and catechins, especially EGCG, were endowed with multiple bioactivities, including antioxidant, anti-inflammatory, immunomodulatory, genoprotective, chemopreventive, skin regenerative, and antilipogenic ones, which could be involved in their healing effects in skin. Particularly, antioxidant properties have been found mediated by radical scavenging mechanisms, along with an increase in endogenous antioxidant defenses, likely due to the activation of the inhibition of the Nrf2 cascade. Anti-inflammatory activity arises from the inhibition of enzymes involved in prostanoid biosynthesis, especially cyclooxygenase 2, downregulation of Nf-kB cascade, modulation of cytokine secretion and of skin cell activation, and increased expression of Tollip protein, which, in turn, block Toll-like receptor signaling. Green tea extracts reduce immune cell infiltration in damaged skin, which denotes immunomodulatory effects, along with the ability to block DNA damage and to modulate apoptosis, leading to genoprotective and chemopreventive effects. Some evidence also displayed their ability to promote sebocyte apoptosis and to block pathways (i.e., MLPK-SREBP-1, PI3K, and mTOR) involved in sebum production. At last, skin-regenerative effects, associated with the ability of green tea phytocomplexes to stimulate elastin and fibronectin production, to control the collagen synthesis, and to improve local blood reperfusion were reported too.

A major limit in the exploitation of green tea and GTP benefits in nutraceutical and cosmetic fields is their low bioavailability, which led studies to develop alternative administration routes and formulations, such as hydrogels or nanoparticles. Particularly, the use of EGCG is limited by lack of stability, especially at high temperatures, which favor its degradation due to oxidation or epimerization to gallocatechin gallate (GCG) [13]. In addition, there are safety concerns about the possible liver toxicity of green tea preparations, in which EGCG seems to be involved, although the true mechanisms remain to be clarified [24,25]. This evidence suggests the need to clarify the true ratio between health benefits and safety issues of green tea consumption, to exploit its healing properties but avoiding unpleasant adverse effects. In this context, the present study was aimed at reviewing clinical evidence about the benefits of the oral administration of green tea preparations and its polyphenols to relieve skin disorders, in order to point out the current knowledge and suggest possible novel strategies to effectively exploit the properties of green tea, also managing safety risks.

## 2. Methodology

To perform the study, a systematic review of the existing literature in PubMed, Scopus, and Web of Science databases was carried out, using PRISMA (Preferred Reporting Items for Systematic Reviews and Meta-Analysis) method [26]. Literature from a 20-year period was considered in May 2022, using the following keywords: “oral green tea”, “tea polyphenols”, “skin”, “erythema”, “dermatosis”, “acne”, “*Camelia sinensis*”, and their combination through the Boolean logical operators. Moreover, clinical trials and randomized clinical trials, in which green tea preparations and green tea polyphenols were administered orally, were selected. Preclinical studies, papers focused on other substances or where green tea was administered topically were excluded. The searches were filtered to include only papers written in English.

## 3. Results

**Figure 4:**
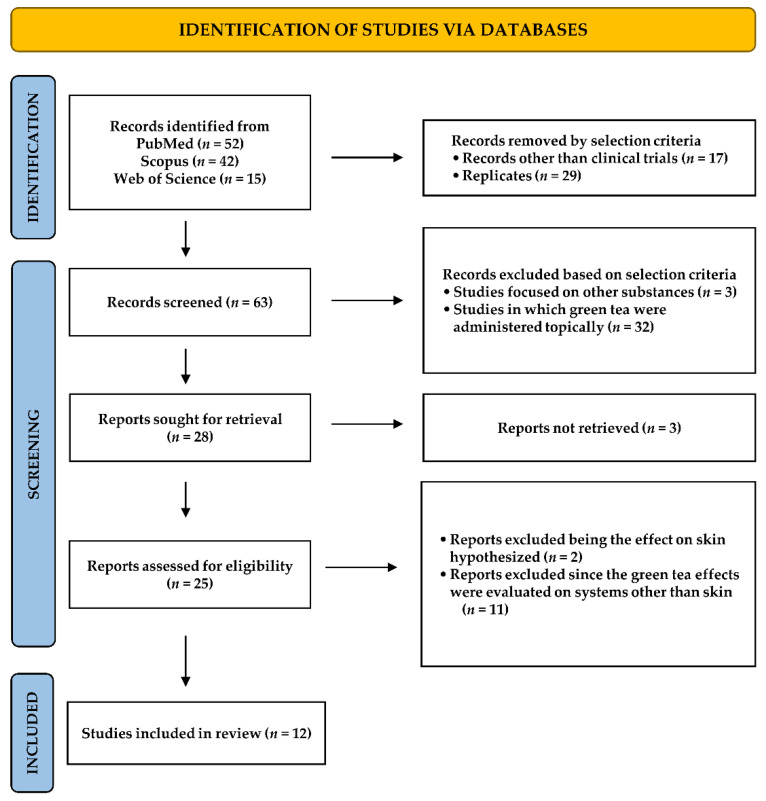
Study selection by PRISMA flow diagram of clinical studies investigating efficacy and safety of oral green tea preparation in skin diseases.

Our literature searching highlighted the presence of a total of 109 published papers focused on clinical trials evaluating the healing properties of green tea preparations and green tea polyphenols in skin diseases (Figure 4). Among the identified studies, 17 papers were removed for being studies other than clinical trials, while 29 were replicated. Out of 63 eligible papers, based on selection criteria, a further 35 papers were excluded: specifically, 3 were focused on other substances than green tea and in 32, green tea was administered topically. We reviewed 28 papers, among which 3 reports were not retrieved, 2 were deleted since the benefit of green tea on skin was just hypothesized, and 11 evaluated the healing properties of green tea on systems other than skin. Based on the PRISMA analysis, a total of 12 studies was included in the systematic review. The major studied conditions were photoaging and UV-induced erythema [27,28,29,30,31,32,33,34]; moreover, some studies evaluated the benefits of different green tea extracts in the treatment of acne, genodermatosis, and in improving antioxidant defenses, skin texture, and integrity in healthy subjects [35,36,37,38]. Usually, decaffeinated green tea preparations were used and compliance of the enrolled subjects was monitored through measuring biochemical parameters. Details on the selected studies are described below and resumed in Table 1.

### 3.1. Photoaging

Photoaging is a type of premature skin aging induced by extrinsic factors, mainly solar radiation, including ultraviolet (UV), visible, and infrared light; more recent evidence also highlighted the contribution of air pollutants to the process, promoting oxidative damage and skin inflammation [39,40]. Fine rhytides, telangiectasias, dyschromias, solar lentigines, leathery skin texture, decreased elasticity, and increased susceptibility are typical signs of photoaging [40,41]. Moreover, skin damage can increase the risk of cell degeneration and cancer development [39]. Regarding mechanisms accounting for photoaging, UV irradiation is known to generate reactive oxygen species (ROS), leading to marked oxidative stress, which impairs the skin functional and regenerative potential. Moreover, activation of photoaging triggers cellular senescence and inflammation, a condition known as inflammaging, and progressively suppresses the skin immune system, increasing susceptibility to infection, degenerative processes, and cancer [42,43]. Molecular targets, including epidermal growth factor (EGF), keratinocyte growth factor, tumor necrosis factor (TNF)-α, and interleukin (IL)-1, are activated by UV radiation, thus, stimulating the transcription factor AP-1, which blocks the synthesis of collagen, while promoting transcription of matrix metalloproteinase (MMP) genes, which in turn triggers imperfect tissue repair and dermal damage [44]. After repeated exposure to sun, dermal damage is accumulated, resulting in wrinkles and photoaging signs [44].

Several studies outlined the role of dietary intake in terms of antioxidant and anti-inflammatory agents to prevent both chronological and solar aging; among them, polyphenols have been highlighted as promising agents in skin health maintenance, due to their antioxidant, anti-inflammatory, and regenerative abilities, likely mediated by the regulation of signaling cascades (e.g., Nrf2, NF-κB), matrix metalloproteinases, and cytokines [45]. This evidence supported the interest in green tea extracts and polyphenols as possible strategies to counteract photoaging.

In a double-blinded, randomized pilot study, Chiu et al. [27] studied the efficacy of an oral green tea preparation in forty healthy women with moderate photoaging (grade 2 to 3 according to Glogau’s classification) and with Fitzpatrick skin phototypes I to III [46]. Particularly, 300 mg of the green tea supplement was taken orally twice a day for 8 weeks, and a 10% green tea extract cream was applied twice a day on the face and arms too. Clinical and histological analysis showed significant improvements in the tissue elasticity of treated specimens but not in clinical grading of photoaging with respect to the placebo group; rather an irritating effect, likely due to the 10% green tea extract cream, was reported. The authors suggested that the 10% green tea concentration is too high and scantily tolerated by skin; therefore, lower amounts should be considered. Moreover, they concluded that, although green tea polyphenols are claimed to protect human skin from photoaging, a 2-month topical and oral supplementation is not sufficient to achieve clinically visible improvements on the skin. Some limitations of the study, including short duration of the treatment and the combination of both oral and topical green tea administration, which hinders clarifying how the protective and regenerative properties of green tea can be exploited, have been discussed: some benefits could require prolonged treatment, thus, being not evaluable under the experimental conditions of this study. Another issue to be considered is the phytochemical composition of the green tea extract, which was characterized only for the catechin content, i.e., 38% EGCG and 14% ECG, along with minor amounts of other flavanols (Table 1); although they have been mainly associated with the bioactivities of green tea, the contribution of other components should be considered too. This represents an important limit of almost all the selected trials, which should be mentioned in order to clarify the possible subtle interactions occurring in the green tea phytocomplex and responsible for its bioactivities. Consequently, an improved standardization for green tea extracts, focused on catechins and other contained compounds, as well as higher reproducibility in healing effects, could be achieved.

The antiphotoaging effects of green tea were also examined by Janjua et al. [28] in a double-blind, randomized, placebo-controlled clinical trial, involving 56 women (aged 25 to 75), who received a 2-year oral supplementation with a green tea extract. The extract was produced by a two-step extraction of tea leaves, using hot (70 °C) water and then ethyl acetate as solvents, and then spray drying the extract and obtain a decaffeinated (<1.3 mg/250 mg extract) powder. High-performance liquid chromatography (HPLC) revealed the presence of about 70% catechins, 38% of which was EGCG and 14% ECG, with lower than 10% of other flavanols [28]. The recruited women had a facial II (moderate) to III (advanced) Glogau grade of photoaging and type I to III Fitzpatrick skin [46]. They received a daily treatment with one capsule, containing 250 mg of green tea extract or placebo: green tea extract dosage corresponded to approximately seven Asian-sized cups of green tea. At zero time and after 6, 12, and 24 months, women were monitored by blinded certified dermatologists and skin histology was evaluated based on 4 mm punch biopsies. Clinical and histological results did not show differences in the photoaging parameters across time points in green-tea-treated subjects, with respect to those receiving placebo. However, a lowering in overall solar damage at 6 months, and in erythema and telangiectasias at 12 months in the green-tea-treated group, but not in placebo, was found. No differences were observed at the 24-month time point. The authors supposed that the registered improvements could be due to changes in sun exposure and activities of involved people, conditioned by their involvement in the study. At any rate, this issue is not supported by evidence, since a sun exposure diary of women was lacking. Another limit discussed by the authors is the lacking evaluation of skin pore size: increased pore size can be induced by sun destruction of elastin and collagen, leading to elasticity loss, but can be prevented by green tea polyphenols, thanks to their antioxidant and anti-inflammatory properties. Further larger studies are needed to clarify the true antiphotoaging benefits of green tea and to define the best conditions to exploit them.

At last, it is interesting to consider that the studied extract was enriched in catechins, one-third of which was EGCG, and used under a prolonged 2-year treatment, according to Chiu et al. [27]. This suggests that, despite the claimed crucial role of catechins as bioactive compounds of green tea, their higher concentration did not improve the antiphotoaging properties. Therefore, other unknown compounds could contribute or lead to the healing effects of green tea; future clarifying studies are recommended.

More recently, Granger et al. [29] evaluated the antiphotoaging and photoprotective efficacy and tolerability of a multicomponent food supplement (VitAoX ultra^®^), containing 10% of an unspecified green tea extract in combination with vitamins A, C, D3, E, selenium, lycopene, lutein, polypodium, and grape extracts, in a 12-week open, prospective, and monocentric clinical study. The study enrolled 30 participants with Fitzpatrick skin type I–III and clinical signs of chronological aging and/or photoaging, which received one capsule of the food supplement (VitAoX ultra^®^) daily for 12 weeks [29]. Treatment resulted in enhanced skin resistance to UV injury, as shown by the increased minimal erythema dose (MED), defined as the lowest dose producing visually discernible erythema; moreover, skin antioxidant capacity and skin parameters (i.e., moisturization, elasticity, radiance, and color of dark spots) markedly improved.

Despite these healing effects being ascribed to the antioxidant power of the product, the true contribution of green tea extract cannot be inferred, since the benefits could be a result of the interactions of multiple components. A further issue to be highlighted is the unknown composition of the green tea extract, along with those of the other extracts, which not only limits the reproducibility of the study but also does not allow one to define the possible bioactive compounds. Using standardized and chemically characterized extracts should be a prerequisite for clinical studies, since it is essential to ensure reproducibility in the outcomes.

### 3.2. UV-Induced Erythema

Ultraviolet (UV) solar radiation (about 6.8% of total radiation) is acknowledged to be responsible for various skin damage, since it can damage lipidic sheets and protein, thus, impairing its water-impermeable barrier function [47]. Indeed, although severity depends on genetic factors, age, nutrition, and exposure, UV radiation causes skin inflammation, DNA damage, and alterations to lipids and proteins, leading to erythema, photoaging, skin lesions, dermatosis, and cancer [48,49]. UVB (280–315 nm) and UVA (315–400 nm) radiation is more dangerous than UVC (<280 nm), which is absorbed in the stratosphere. UVB radiation directly affects DNA integrity, by forming covalent links between adjacent pyrimidine bases (photodimers), thus forming cyclobutane pyrimidine dimers and pyrimidine-6,4-pyrimidone photoproducts; by contrast, UVA (315–400 nm) produces indirect genotoxicity, due to the generation of reactive oxygen species (ROS), which, in turn, damage DNA and other biomolecules [50].

Several natural compounds, especially flavonoids, flavanols, and carotenoids, have been found endowed with photoprotective effects, likely due to their antioxidant, anti-inflammatory, and immunomodulatory properties [51]. In line with this evidence, some clinical trials have been carried out to evaluate the clinical efficacy of oral preparations of green tea to prevent skin damage induced by UV radiation.

Heinrich et al. [30] investigated the effects of repetitive intakes of a green tea beverages on skin resilience to UV radiation, along with improvements in texture and microcirculation, in a double-blinded, placebo-controlled trial, involving a total of 45 females (40–65 years old) with type II Fitzpatrick skin [46]. Particularly, 30 subjects received an oral administration of a green tea beverage (1 L daily, corresponding to 1402 mg total tea catechins, half of which was ECGC) or a control beverage (containing a non-nutritive sweetener, ascorbic acid, and quinine hydrochloride to mime the bitter taste of green tea) for 12 weeks. Before starting the study, back and scapular regions of women were irradiated with 1.25 MED (minimal erythemal doses), using a blue-light solar simulator. At baseline (zero time) and after 6 and 12 weeks, blood samples were drawn to measure the blood flavanol levels and skin variables (i.e., elasticity, structure and texture, hydration, reddening) were evaluated.

A significant 16 to 25% lowering of UV-induced erythema, along with increased plasma levels of EGCG, followed by EGC, EC, and ECG, were highlighted in the green tea group, with respect to the control; moreover, skin elasticity, hydration, texture, and structure were improved, thus, suggesting the ability of the treatment to protect skin against UV injury and to boost its functions. These benefits were associated with an increase in blood flow and oxygen delivery to the skin, which can result in improved nutrient and oxygen supply to it, improving the skin condition and appearance.

These benefits were also confirmed, in a second short-term randomized, double-blinded trial by Heinrich et al. [30], involving 15 female volunteers not included in the previous one, divided into subgroups, which received a single dose of 0.5, 1.0, or 2.0 g of a green tea SP 90 DCF-T extract (formulated in capsules containing 0.5 g extract). At HPLC analysis, the extract was found concentrated in catechins, of which at least 50% was EGCG. At 30 min after the SP 90 DCF-T extract administration, a transient but not dose-dependent alteration in blood flow occurred and levels of serum epicatechin were enhanced. According to previous evidence, the authors suggested that the known abilities of polyphenols and flavanols to absorb UV light and to counteract oxidative stress, inflammation, and DNA damage [52] represent the major mechanisms accounting for their photoprotective effects and can contribute to the healing effects of green tea preparations against UV radiation damage. The extent of UV protection by green tea is comparable to that reported for polyphenol-based cocoa extracts [53]; however, the specific contribution of green tea flavanols remains to be clarified. Altogether, Heinrich et al. [30] highlighted the ability of both beverage and extract from green tea to produce protective effects on skin from UV injury and to support skin structure and function.

In line with these findings, Rhodes and colleagues [31] performed a pilot open uncontrolled study, in which they studied the photoprotective effects, mediated by a reduction in pro-inflammatory eicosanoid production, of a green tea extract (containing 40% catechins, 40.3% of which was EGCG while 27.4% was EGC and 14.4% ECG). Three capsules containing the green tea extract were administered orally, in association with two capsules of vitamin C (total 1350 mg tea green extract, corresponding to 540 mg catechins, plus 50 mg vitamin C daily), to sixteen white healthy subjects with sun-reactive skin type I–II for 12 weeks. As declared by the authors, vitamin C was needed to stabilize green tea polyphenols in the gut lumen [54], without an impact on UV-R erythema [55]. UV damage skin erythema was induced by a solar simulator (source of UVB and UVA radiation); compliance was measured by the analysis of polyphenols in urine samples, collected during treatment.

The results showed that the treatment suppressed UV-induced erythema and skin inflammation; indeed, a lowering in the rise of proinflammatory factors, i.e., 12-HETE eicosanoid and PGE_2,_ was found. Skin biopsy samples also highlighted increased levels of catechins in tissue, thus, suggesting a possible involvement of these compounds in the healing effects of green tea; however, the contribution of other phytochemicals in the mixture, along with that of vitamin C, cannot be excluded. Rhodes et al. [31] also showed the ability of green tea supplementation to regulate the UV-induced release of lipoxygenase metabolite and leukocyte chemoattractant 12-HETE, alongside cutaneous leukocyte infiltration; this last effect was also reported after topical application of green tea preparations. Owing to the pivotal role of UV radiation in skin cancer development, likely acting as a genotoxic agent, and considering the oncogenic role of 12-HETE and its overexpression in many cancers, including skin cancers, the ability of green tea supplementation to affect its biosynthesis suggests a possible chemopreventive interest, which deserves further confirmation studies.

In line with the previous promising evidence, the research group in [32] carried out a further 12-week double-blind, randomized, placebo-controlled trial, involving fifty healthy white adults (both male and female; aged 18–65 years), carrying I to II Fitzpatrick sun-reactive skin types, to evaluate the photoprotective effects of green tea preparations. Recruited subjects received a twice daily treatment with three gelatin capsules of a green tea supplement (total 2700 mg green tea extract, containing 40% catechins (corresponding to 1080 mg), 16.13% of which was EGCG, while 10.96% and 5.78% were EGC and ECG, respectively) plus two gelatin capsules of vitamin C (100 mg), or placebo gelatin capsules with maltodextrin, for 12 weeks. Unexpectedly, they found lacking effects of the combined treatment of green tea extract/vitamin C on the skin erythema, dermal leukocytic infiltration, and inflammatory response induced by UV exposure. The differences were discussed by the authors, highlighting some limitations of the previous pilot study, especially the enrollment of nearly all females, with possible sex-specific effects and the inability to measure effects unrelated to green tea supplementation; therefore, Farrar et al. [32] suggested the need for further studies to better elucidate the healing effects of green tea extracts and catechins.

In a following double-blind, randomized, placebo-controlled study [33], the photoprotective effects of the green tea extract/vitamin C supplementation were evaluated in healthy adults, with Fitzpatrick skin phototypes I and II, against a high-proinflammatory UV dose (3 × minimal erythema dose MED), emitted by a solar simulator (5% UVB, 95% UVA). Particularly, fifty subjects (13 males and 37 females; 18–65 years of age) took a 1080 mg green tea extract (equivalent to 5 cups of green tea beverage) with 100 mg vitamin C daily, or placebo (i.e., maltodextrin), for 12 weeks. The rate of cyclobutane pyrimidine dimers (CPD) in skin, which is typical DNA damage induced by UV radiation, was measured by immunohistochemical staining analysis. Results highlighted neither difference in UV-induced DNA-damage (in term of CPD) between green tea and placebo groups, nor within groups at baseline and post-supplementation. However, the presence of epigallocatechin glucuronide in urine confirmed the compliance of the recruited subjects. The authors discuss these conflicting results, with respect to the evidence of genoprotective effects of green tea treatments in topical human studies [56]; however, they suggest that higher local skin concentrations, with respect to that achieved after ingestion, could be responsible for the different outcomes and remark the need for more studies to elucidate the best conditions and features to exploit the possible benefits of green tea preparations against UV radiation damage.

Despite the negative results of Farrar et al. [32,33], a recent systematic review of the literature followed by a metanalysis by Kapoor et al. [57] showed that oral green tea supplementation, both low and high doses, significantly protects against low-dose ultraviolet-radiation-induced erythema response, with less efficacy against high-intensity UV. Topical sunscreens, which are effective for a finite time, require frequent application and do not protect cutaneous immunity; oral supplementation can provide a constant low level of photoprotection to the entire body surface and strengthen the skin’s tolerance to UV radiation damage; therefore, it can be exploited as adjuvant or alternative sunscreen-protective intervention.

These benefits were confirmed by a recently published double-blind randomized clinical trial by Charoenchon et al. [34], evaluating the ability of the treatment green extract plus vitamin C against changes in the dermal collagen and elastic fiber systems, following exposure to solar-simulated radiation (SSR). Particularly, 50 healthy white adults (aged 18–65 years), carrying Fitzpatrick skin types I–II, took a total daily dose of 1080 mg green tea extract and vitamin C 100 mg, as gelatine capsules, or placebo (i.e., maltodextin), and were exposed to SSR for 12 weeks; compliance of the subjects was confirmed by measuring the urine levels of catechins. The results highlighted that green tea supplementation protected fibulin-5, and potentially fibulin-2, against SSR-induced changes in the components of dermal collagen and elastic fiber in human skin; these effects could be ascribed to the ability of epigallocatechin gallate to attenuate elastic fiber degradation by matrix metalloproteinase [58], while the contribution of vitamin C seems unlikely based on the available evidence [59]. These findings strengthen the need for future studies to evaluate the possible protective role of green tea supplementation on human skin against repeated suberythemal UV-R exposure.

### 3.3. Acne Treatment

Acne vulgaris is a skin disorder developed as a consequence of multiple factors, including hormonal changes, stage of life, nutrition, medications, exposure to pollutants, climatic factors, and infections [60]. It is typical of adolescence, although it can be manifested during other life stages, such as neonatal and late acne. Key factors in the development of acne are an increased or altered sebum production by piliferous glands (hyperseborrhea and dysseborrhea), changes in keratinization, skin inflammation, and infection of piliferous glands by *Propionibacterium acnes*; moreover, changes in hormone secretion, particularly androgens, insulin, and insulin-like growth factor-1, play a centrale role too [61,62]. The common treatment of acne often includes combinations of oral and topical antimicrobials and retinoid drugs, hormonal agents, along with chemical peeling and laser therapy; however, relevant side effects, such as microbial resistance, cheilitis, and teratogenicity, have been reported, leading to the need to discover alternative therapeutic strategies [63]. From this perspective, green tea preparations for both topical and systemic administration appeared promising strategies due to their antimicrobial, anti-inflammatory, antioxidant, and regenerative properties.

Our literature searching revealed the presence of a randomized, double-blind, placebo-controlled clinical trial, which investigated the efficacy and safety of an oral preparation containing a decaffeinated green tea extract (GTE) in women carrying post-adolescent acne [35]. GTE extract (provided by Tea Research and Extension Station, Taoyuan County, Taiwan) was standardized to contain about 57% EGCG and 16% ECG, with minor levels of other catechins, administered by capsules (i.e., 500 mg extract per capsule). To perform the study, 80 subjects were randomly assigned to two groups, receiving 1500 mg of GTE or placebo (cellulose) daily for 4 weeks, and monitored for inflammatory lesions associated to acne at zero time and at the end of treatment. A significant lowering in the number of acne lesions on the nose, perioral area, and chin—but not in the total lesions—was highlighted in the GTE group after 4 weeks of treatment, thus, suggesting possible benefits of green tea intake in acne control [35]. However, some limitations of the study, including the small sample of recruited women and the large variance in lesion counts, weaken the clinical evidence.

Despite limited evidence in support of the oral use of green tea for acne, other studies highlighted the efficacy of topical formulations containing green tea extracts (2% lotion or 3% emulsion alone or with other treatments, ointment) to relief acne lesions, with only minor side effects [64,65,66,67,68]. Yoon et al. [22] also reported significant benefits in 35 acne patients after 1% and 5% EGCG topical treatment for 8 weeks. Similarly, Waranuch et al. [69] highlighted the antiacne and antiblotch efficacy of a hydrogel, containing a combination of *Aloe barbadensis* leaf extract, *Garcinia mangostana* peel extract, and *Camellia sinensis* leaf extract (50:25:1), with respect to a 1% clindamycin gel. However, these studies had a limited methodological quality, due to a low number of enrolled subjects, control groups, or lacking randomization. Therefore, novel high-quality studies are expected to clarify the interest in green tea and its polyphenols as anti-acne oral treatments.

### 3.4. Genodermatosis

Genodermatosis refers to alterations in skin function associated with genetic mutations, some of which are caused by nonsense mutations that trigger the production of truncated or non-functional proteins. Among them, recessive dystrophic epidermolysis bullosa (RDEB) is a rare genodermatosis characterized by mutations in the COL7A1 gene, which leads to disorders of collagen production and skin fragility, increasing the risk of wounds and scarring [70]. Usually, treatments are mainly symptomatic, with no cure [70]: this strengthens the interest in alternative strategies, such as nutraceuticals or natural substances, to improve health status and life quality in patients. Chiaverini et al. [36] carried out a multicenter, randomized, crossover, double-blind, placebo-controlled clinical trial, in which the efficacy and safety of a green tea extract was evaluated in 17 RDEB patients after a 4-month oral treatment. Although not detailed by Chiaverini et al. [36], the product used in the clinical study (Clinical Trial Register NCT00951964) was a standardized green tea extract (Polyphenon E^®^), containing about 65% EGCG, along with minor levels of other catechins [71]. Recruited subjects received the treatment (corresponding to 400 to 800 mg EGCG daily, on the basis of the patient’s weight) or a placebo. Despite a slightly increased wound healing and lowered frequency of new blisters, the treatment did not produce relevant benefits in RDEB patients with respect to the placebo. These conflicting results are discussed by the authors in light of the study limitations, especially the low number of enrolled subjects, along with differences in the severity of phenotype and sensibility to treatment. Therefore, they suggest the need for a future international randomized, double-blinded, and placebo-controlled trial, with a targeted subpopulation to clarify the possible benefits of green tea preparation in genodermatosis.

### 3.5. Skin Integrity and Antioxidant Defenses

The major function of human skin is to protect from exogenous injuries, including mechanical damage, chemicals, pathogens, and radiation; moreover, it regulates the excretion of metabolic waste and homeostasis of body temperature [72]. Acting as a barrier, it is directly exposed to the injury of many chemical, physical, and biological toxicants, especially free radicals, UV radiation, and environmental pollutants, which often induce oxidative stress and damage of polyunsaturated fatty acids, leading to altered function and diseases, such as acne, psoriasis, and aging. Maintenance of skin homeostasis is guaranteed by the presence of antioxidant skin defenses and regenerative abilities, which could be impaired during marked oxidative stress and injury. Therefore, boosting skin defenses, especially its modulating antioxidant status and integrity, through using nutraceuticals, dermatological protective agents, and dietary supplements, could represent a suitable strategy to activate skin resilience and prevent the development of diseases.

Chiu et al. [37] carried out a randomized, crossover, double-blind, placebo-controlled clinical trial, involving 44 volunteers (25 to 80 years old), who consumed 240 mL of mineral water mixed with either green tea preparation or placebo (two packs daily at morning and evening) every day for 6 months and vice versa, with one month of washout. Green tea packs contained an undefined green tea preparation with milk powder (named green tea polyphenol milk or GTPM), skim milk, whey protein, lactoferrin isolated from soy, and other supplements (e.g., maltodextrin, vegetable oil, sugar, minerals, vitamins, and carrageenan), while the placebo packs contained all the ingredients except for green tea. GTPM supplementation was found to ameliorate skin texture and integrity, to lower skin wrinkles and roughness in elderly subjects, and to increase total phenolic content in plasma, antioxidant index, and enzyme activities, with respect to the placebo group. These findings suggested that milk proteins increased the absorption of polyphenols, leading to increased plasma levels and improved antioxidant capacity, which likely contribute to the skin quality amelioration. Results of other studies agree with the increased polyphenol absorption triggered by milk [73,74], although opposite results are reported too [75,76]. Despite limitations in this pilot study, due to lacking information about green tea extract and its catechin content, along with those declared by the authors, i.e., limited number of recruited subjects, lacking GTP (green tea preparation without milk) control group, and determination of catechin plasma levels, the results suggest an interest in green tea preparation combined with milk to stimulate antioxidant defenses and improve skin quality.

Increased skin radical scavenging properties after the consumption of Japanese Benifuuki and Yabukita green teas were also highlighted by Megow et al. [38] in a randomized clinal trial involving thirty-two participants. Subjects were asked to consume three cups (total 600 mL) per day of either Benifuuki tea or Yabukita tea, or water (control group), for 2 weeks. At the end of the study, analysis by electron paramagnetic resonance (EPR) spectroscopy was made to monitor the antioxidant status of human skin after oral green tea administration and to compare the cutaneous radical scavenging effects of Benifuuki and Yabukita teas. The results revealed that the skin radical scavenging properties, with respect to the control, were increased by 28 and 29% in the Yabukita and Benifuuki green tea groups, respectively, without changes in total carotenoid content. Although methylated catechins of Benifuuki tea are reported to be more bioavailable, no difference in the outcomes of the Yabukita and Benifuuki tea groups was highlighted. The role of green tea polyphenols and their metabolites in the potentiation of antioxidant skin power remains to be clarified in future studies. Another issue to be considered is the lowered effect of green tea supplementation in subjects affected by illness during the study; this could be a result of an impairment in cutaneous antioxidants due to illness, as previously reported. However, as discussed by authors, the lacking monitoring of catechin levels in blood or urine enables one to exclude that non-responder subjects were noncompliant.

Altogether, results of the study by Megow et al. [38], highlighting the ability of orally administered green tea to improve the skin antioxidant status, suggests its possible usefulness in the prevention of oxidative-based skin injury and diseases, such as premature skin aging, skin cancer, psoriasis, atopic dermatitis, and acne. However, owing to limitations in the available studies, i.e., lack of blinding of the control group, small sample size, and short duration, larger high-quality trials are needed in confirmation.

### 3.6. Safety Evaluation

Selected clinical trials usually highlighted that oral green tea preparations, administered usually for 8 to 16 weeks, were well tolerated with minor ailments. This is also confirmed in the 2-year treatment reported by Janjua et al. [28], which highlighted similar nonserious adverse events in green tea and placebo groups, among which, upper respiratory tract infection followed by loose stools and other minor gastrointestinal ailments were the most common. Two cases of serious adverse events, including appendicitis and retinal detachment, occurred during treatment with green tea preparation but the causality relationship remains unclear [28]. However, the available literature did not support the association of green tea ingestion with these adverse effects; by contrast, regarding retinal detachment, some preclinical evidence suggests the possible protective role of green tea polyphenols against cellular toxicity of the retina [77].

Similarly, the ailments reported by two subjects involved in the study of Granger et al. [29], i.e., slight stomach burns and difficulties of digesting the product, were not clearly related to the green tea supplementation. Chiu et al. [27] did not describe specific side effects due to oral treatment with green tea; conversely, irritation and sun sensitivity were caused by topical application of the 10% green tea cream, thus, suggesting that it represents a too great concentration, which is to be avoided. Occasional mild nausea after ingestion was also reported by Rhodes et al. [31] and Farrar et al. [32] after ingestion of the green tea supplements, although it was usually well tolerated by all the other recruited subjects. Accordingly, Lu et al. [35] registered one subject who developed mild constipation and two others with abdominal discomfort after treatment, but without facial ailments and major adverse effects in all the involved subjects. A good tolerability was also reported for the green tea Polyphenon E^®^ extract, although some cases of gastroenteritis, vomiting, odynophagia, esophageal blister and pain, constipation, pruritus, asthenia, and bronchitis were reported [36]. Similarly, Mengow et al. [38] highlighted symptoms of illness (usually common cold) in some of the subjects receiving Yabukita and Benifuuki green tea beverages. Conversely, tolerability data in the studies by Heinrich et al. [30], Farrar et al. [33], Charoenchon et al. [34], and Chiu et al. [37] were lacking. This evidence strengthens the need to clarify the causality relationships of the mild and occasional adverse effects occurring in clinical trials, especially gastro-intestinal ailments, and the use of oral green tea preparations. To this end, specific pharmaco-toxicological and phytochemical evaluations should be encouraged in order to understand the mechanisms of action and the contribution of green tea phytochemicals.

Despite the generally good tolerability of green tea oral preparations used in the selected clinical trials, green tea is the hub of a lively debate on pros and cons of its use as a dietary supplement, due to well-documented case reports of adverse events, especially hepatotoxicity, associated with the consumption of green tea beverages and supplements, alone or in combination with other products. Liver damage usually occurred when the products were taken for weight loss, although some cases in which the supplement was used to counteract hair loss and for improving well-being have been reported too [24,25]. Accordingly, two recent hepatotoxicity cases associated with green tea consumption have been reported too [78]. The suspected products often were characterized by a different catechin composition and sometimes EGCG high-concentrated extracts were used; in many cases, green tea preparations were taken in combination with other components, including botanicals, pure phytochemicals, vitamins, amino acids, and others. The duration of treatment was variable from days to more than 1 year; similarly, adverse reactions (sometimes severe, requiring hospitalization and liver transplantation) occurred quickly or after a long-term exposure [24,25]. The causality assessment highlighted a probable or possible relationship between green tea consumption and hepatic adverse reaction, although the mechanisms of hepatotoxicity remain unclear and the contribution of other factors, such as genetic features, individual susceptibility, idiosyncrasy, and concomitant use of other products, seems to play a pivotal role in adverse reaction development [24,25]. Among the possible contributing factors, some evidence highlighted that fasting significantly increases the bioavailability of catechins, especially that of EGCG, likely leading to saturation of first-pass elimination mechanisms and toxicity development. Based on these findings, the United States Pharmacopeia (USP) monograph on “Powdered Decaffeinated Green Tea Extract” includes the following cautionary labeling requirement: *“Do not take on an empty stomach. Take with food. Do not use if you have a liver problem and discontinue use and consult a healthcare practitioner if you develop symptoms of liver trouble, such as abdominal pain, dark urine, or jaundice (yellowing of the skin or eyes).”* [25]. Accordingly, the European Food Safety Authority (EFSA) declared that, based on clinical studies, EGCG levels equal or above 800 mg daily can affect liver function; however, considering that sometimes hepatotoxicity occurred at lower doses, a true safe EGCG dose cannot be established [79]. Therefore, it is important to follow the recommendations by Mazzanti et al. [24], i.e., using high-quality standardized green tea extracts (lacking risk of contamination, adulteration, and misidentification), to avoid multiple co-administration, since green tea components can affect both metabolic and excretion processes of drugs, with an increased risk of interactions and toxicity, and to discourage green tea use in subjects with liver ailments or with impaired liver functions, and further, when a greater susceptibility to adverse reactions could be expected [24]. Furthermore, more specific pharmaco-toxicological studies should be encouraged to clarify the currently unknown toxicity mechanisms and to likely identify possible high-susceptible people [24]. At last, the supervision of qualified healthcare professionals should be recommended, in order to promptly highlight possible sensitive subjects at risk of adverse effects [24].

## 4. Conclusions and Future Perspectives

Green tea preparation and its polyphenols have attracted great attention over the years as possible nutraceutical agents due to their promising antioxidant, anti-inflammatory, chemopreventive, and immunomodulatory properties, which could be exploited for several diseases, including skin ailments. Preclinical studies suggested that in some skin disorders, such us UV-induced erythema, acne, or photoaging, these properties, along with regenerative and genoprotective effects, could be advantageous to relieve skin integrity and texture and to stimulate endogenous defenses [80], thus, leading to tissue regeneration and damage repair.

Nevertheless, the available clinical trials focused on oral green tea preparations are only few: we selected a total of 12 studies, among which 5 focused on UV-induced erythema and skin alterations, 3 on photoaging, 2 on antioxidant skin defenses, while only 1 study for acne and genodermatosis. These trials highlighted several benefits of green tea preparations in skin ailments, usually ascribed to the ability of green tea polyphenols to counteract oxidative stress and inflammation induced by exogenous stimuli, to promote tissue regeneration and endogenous skin antioxidant defenses, and to modulate immune system infiltration.

On the basis of these findings, we can conclude that the evidence supports the use of oral green tea preparations to protect skin from damage induced by low-dose ultraviolet radiation; however, a higher than 2-month supplementation seems to be needed for clinically visible improvements on the skin. Therefore, application of oral green tea preparations as adjuvant or alternative sunscreen-protective interventions could be encouraged, in compliance with the safety recommendations. The other studied conditions provide only preliminary evidence due to conflicting results, as shown in photoaging, or limited low-quality studies, as highlighted in the cases of acne, genodermatosis, and for the boosting effects on antioxidant status and skin integrity.

The major limits of the selected studies are small sample size of enrolled subjects, short duration of the study, combination of both oral and topical green tea administrations, combination of green tea with other products, and lacking or scant standardization of green tea extract. In this respect, it should be remarked that usually, the authors refer to green tea polyphenols, green tea catechins, or even to EGCG, while they used a green tea extract, usually containing 40% to 70% catechins.

Although catechins are considered the major bioactive compounds in green tea, the contribution of other components cannot be excluded. This is an important issue to be considered since the fraction of unknown compounds can contribute both positively and negatively to the whole effects, and their uncontrolled variation in the tested extracts could contribute to achieving conflicting outcomes. Therefore, an improved standardization and a much more complete phytochemical characterization of green tea extracts could represent a future strategy to improve reproducibility of the green tea healing effects, in order to effectively exploit them in therapy. In support, EGCG-enriched extracts were found not effective after a long-term treatment [28].

Many clinical studies also highlighted the clinical benefits of topical green tea preparations, such as ointments, creams, gels, and lotions (containing usually 2% to 15% green tea extracts) [13,14,81,82]; these studies were excluded from this systematic review, since they did not accomplish the inclusion criteria. Particularly, a bath therapy based on a 5% solution of green tea extract produced benefits in patients with atopic dermatitis associated with *Malassezia sympodialis* infection [82]; moreover, a 2% green tea lotion decreased inflammatory lesions in acne subjects [14]. Polyphenon^®^ E formulations (15–10%) have been reported as efficacious for treating external anogenital warts (EGWs), which are non-malignant skin tumors associated with human papillomavirus infection, and for preventing recurrence [81]; accordingly, topical treatment with a sinecatechins 10% ointment (Veregen^TM^) was effective to treat recalcitrant facial warts without surgery [83].

Promising evidence also suggested UV-protective effects of topical green tea formulations, skin wound repair, and chemopreventive benefits in skin cancer [21,84,85,86,87,88]. However, no clinical benefits were found for a topical sinecatechin 10% ointment (Veregen^®^) in the treatment of superficial basal cell carcinoma [89]. Other studies also reported synergistic or additive skin benefits of topical green tea preparations in combination with other herbal extracts, such as lotus, ginkgo, and capsicum, although the true contribution of each component in the mixture was not clarified [90,91,92].

Notwithstanding this promising evidence, topical treatments are limited due to a finite-time efficacy, thermal instability, especially for sunscreen formulations, need for frequent applications, and a lack of protective effects on cutaneous immunity. Conversely, oral supplementation can provide complete and lasting effects, achieving constant outcomes and also boosting endogenous skin defenses; therefore, they could be exploited for several skin ailments, such as photoprotection, cancer chemoprevention, improvement in skin’s resilience and integrity, and for treating skin diseases (i.e., atopic dermatitis, warts, acne). Further studies should be encouraged in order to strengthen the clinical evidence about the benefits of oral green tea preparations in dermatological diseases and to clarify the safety risks and tolerability.

## Figures and Tables

**Table 1:** Clinical trials on the effects of green tea polyphenols in skin diseases.

Authors, Year [Ref.]	Study Design	Participants/N. (Years)	Treatment (Dosage) Duration	Product/Composition	Endpoints	Outcomes	Adverse Events
*Photoaging*							
Chiu et al., 2005 [27]	R, DB, PC	Moderate photoaging/ 40 healthy women	Green tea supplement (300 mg) plus 10% green tea cream twice daily 8 weeks	Undefined decaffeinated green tea extract/38% EGCG, 14% ECG, 7% EC, 6% EGC, 4% GCG, 1% C, <0.5% caffeine	Evaluation of clinical and histological parameters of skin photoaging	Improved skin elasticity	Local irritation due to 10% green tea cream
Janjua et al., 2009 [28]	R, DB, PC	Moderate to advanced photoaging/56 (25–75)	Green tea supplement (250 mg) daily2 years	Undefined decaffeinated green tea extract/70% catechins (38% EGCG, 14% ECG, 7% EC, 6% EGC, 4% GCG, 1% C) and <0.5% caffeine	Clinical and histological parameters of skin photoaging	Lacking effects	Like placebo nonserious events. 2 cases of nonrelated serious events (i.e., appendicitis and retinal detachment)
Granger et al., 2020 [29]	Open prospective and monocentric	Moderate photoaging/30 (40–65)	Multicomponent food supplement/one capsule twice daily 12 weeks	VitAoX ultra^®^ formula/50 mg *Camellia sinensis* L. Kuntze extract, 480 mg *Polypodium leucotomos* Poir, 10.1 mg *Vitis vinifera* L. extract, 40 mg vitamin C, 12 mg vitamin E, 5 µg vitamin D3, 41.5 µg selenium (sodium selenite), 800 µg vitamin A, 8 mg lycopene and 8 mg lutein per capsule	Minimal erythemal dose (MED), antioxidant capacity, skin parameters of aging and treatment tolerability	↑ MED, improved antioxidant capacity and aging parameters	2 cases of not clearly related ailments (i.e., slight stomach burns, digestive difficulties)
*UV-induced erythema*							
Heinrich et al., 2011 [30]	R, DB, PC	Healthy/ 60 (40–65)	Green tea beverage/1 L daily (1402 mg GTC) 12 weeks	Beverage/1402 mg total catechins (70% EGCG, 17% ECG, 7.1% EC, 3.1% GCG, 1.6% C, 0.6% CG, 0.4% EGC, 0.2% GC) and 119 mg ascorbic acid per L	Photoprotection (reddening), skin function and structure, skin blood flow and catechin serum levels	↓UV-induced erythema Improved skin texture and structure ↑ Microcirculation and catechin serum levels	Lacking data
	R, DB, PC	Healthy/ 15 (40–65; 5 people per dose-treatment)	Decaffeinatedgreen tea extract (500 mg capsule)/0.5, 1.0, or 2.0 g daily 12 weeks	Sunphenol 90 decaffeinated (SP 90 DCF-T)/66.5% total catechins (51% EGCG, 8% EC, 4% EGC, 2.3% GC, 1.2% C)	Skin blood flow and catechin serum levels	Transient but not dose-dependent alteration of blood flow and ↑ epicatechin serum levels	Lacking data
Rhodes et al., 2013 [31]	Open intervention study	Healthy/ 16	Oral green tea supplement plus vitamin C/1350 mg green tea extract (corresponding to 540 mg catechins) plus 50 mg vitamin C daily 12 weeks	Undefined green tea extract (450 mg per capsule)/40% catechins (40% EGCG, 27% EGC, 14% ECG, 6.9% EC, 6.9% GC, 2.5% GCG, 1.2% C, 0.2% GA, 0.2% CG)	UV skin sensitivity	↓ UV-induced erythema	4 cases of mild nausea after ingestion
Farrar et al., 2015 [32]	R, DB, PC	Healthy male and female/ 50 (18–65)	Oral green tea supplement plus vitamin C/ 2700 mg green tea extract (corre-sponding to 1080 mg catechins) plus 10 mg vita-min C daily 12 weeks	Undefined green tea extract (450 mg per capsule)/40% catechins (16% EGCG, 11% EGC, 14% ECG, 6.9% EC, 6.9% GC, 2.5% GCG, 1.2% C, 0.2% GA, 0.2% CG)	UVR-induced inflammation	Lacking effects	Mild nausea after ingestion
Farrar et al., 2018 [33]	R, DB, PC	Healthy male and female/ 50 (18–65)	Oral green tea supplement plus vitamin C/ 2700 mg green tea extract (corre-sponding to 1080 mg catechins) plus 10 mg vita-min C daily 12 weeks	Undefined green tea extract (450 mg per capsule)/as for [32]	UVR-induced inflammation	Lacking effects	Lacking data
Charoenchon et al., 2022 [34]	R, DB, PC	Healthy/ 50 (18–65)	Oral green tea supplement plus vitamin C/ 2700 mg green tea extract (corre-sponding to 1080 mg catechins) plus 10 mg vita-min C daily 12 weeks	Undefined green tea extract (450 mg per gelatin capsule)/as for [31]	UVR-induced inflammation	UVR protection to fibulin-5	Lacking data
*Acne*							
Lu et al., 2016 [35]	R, DB, PC	Women carrying moderate to severe acne 80 (25–45)	Decaffeinated green tea extract/1500 mg daily 4 weeks	Undefined green tea extract (500 mg per capsule)/90% catechins (57% EGCG, 16% ECG, 8% EGC, 5% EC, 4% GCG, GC) and <0.07% caffeine	Inflammatory lesion counts	↓ nose, perioral, and chin lesionsTotal lesion unaffected	One case of mild constipation and two with abdominal discomfort
*Genodermatosis*							
Chiaverini et al., 2016 [36]	R, DB, PC, crossover	RDEB patients/ 17 (19.4 ± 16.2 SD)	Green tea extract/400 to 800 mg daily based on body weight 4 months	Polyphenon E^®^ green tea extract (200 mg per capsule)/65% EGCG,9% EC, 6% ECG, 4% EGC, 4% GCG, 0.2% CG, 0.2% GC, 1.1% C, 0.7% caffein	Improvement of RDEB	Lacking effects	Some cases of gastroenteritis, vomiting, odynophagia, esophageal blister and pain, constipation, pruritus, asthenia and bronchitis
*Skin integrity and antioxidant defenses*							
Chiu et al., 2016 [37]	R, DB, PC crossover	Healthy subjects/ 44 (25–80)	Green tea supplement plus milk/2 packs daily 6 months treatment + 6 months placebo	GTPM (green tea polyphenol milk)/131.4 ± 9.2 mg TP ^a^ and 91.7 ^b^ ± 0.5 mg TF per mg dry extract	Skin integrity in relation to oxidative status	↑ skin integrity and texture ↑ antioxidant index ↓ lipid peroxidation	Lacking data
Megow et al., 2017 [38]	R, PC	Healthy male and female/ 37 (20–55)	Freshly prepared green tea beverages/600 mL daily (corresponding to 6 g tea leaves) 2 weeks	Benifuuki and Yabukita teas (lacking chemical characterization)	Skin radical scavenging activity	↑ Skin radical scavenging activity	Symptoms of illness (usually common cold)

R, Randomized; DB, Double-Blind; PC, Placebo-Controlled; GTC, Green Tea Catechins; ↑, Increase; ↓, Reduction; RDEB, Recessive dystrophic epidermolysis bullosa; EGCG, Epigallocatechin gallate; EGC, Epigallocatechin; ECG, Epicatechin gallate; EC, epicatechin; GC, gallocatechin; GCG, gallocatechin gallate; C, catechin; GA, gallic acid; CG, catechin gallate; GTPM (green tea poly-phenol milk. ^a^ TP, total phenolics expressed as gallic acid equivalents. ^b^ TF, total flavonoids expressed as quercetin equivalents.

## Data Availability

Not applicable.
